# Oncocytic Mucoepidermoid Carcinoma of the Parotid Gland

**DOI:** 10.1007/s12105-025-01768-0

**Published:** 2025-03-15

**Authors:** Prokopios P. Argyris, Paul E. Wakely Jr

**Affiliations:** 1 Division of Oral and Maxillofacial Pathology, The Ohio State University College of Dentistry, Postle Hall, Room 2191, 305 W. 12th Ave, Columbus, OH USA; 2 Department of Pathology, The Ohio State University Wexner Medical Center, James Cancer Hospital and Solove Research Institute, Columbus, OH USA

**Keywords:** Oncocytic, Mucoepidermoid carcinoma, Salivary duct carcinoma, MAML2, p63/p40, Androgen receptor, Salivary gland neoplasm

## Abstract

**Case presentation:**

A 30-year-old man presented with a multilobulated left parotid mass measuring 5.2 × 4.1 × 2.4 cm by imaging, and numerous enlarged left cervical lymph nodes, suspicious for metastasis. FNA cytopathology of the mass showed loose clusters of large cells displaying increased N/C ratios and ample granular oncocytic cytoplasm. A superficial left parotidectomy with radical resection of the cheek and cervical lymphadenectomy was performed. Histopathologic examination disclosed a circumscribed, unencapsulated neoplasm exhibiting a solid growth pattern composed of infiltrative islands and nests of cohesive, polygonal, oncocytoid cells in a densely fibrous stroma. Lesional cells exhibited enlarged, oval, open-face nuclei with coarse chromatin and a single acidophilic macronucleolus, voluminous eosinophilic granular cytoplasm and distinct cell membrane borders. Mitotic activity and necrosis were absent. Microcystic architecture was noted solely in a single tumor nest at the periphery. These spaces contained mucinous secretions and were lined by cuboidal oncocytic and intermediate cells with interspersed mucocytes, highlighted by mucicarmine stain. Immunohistochemically, oncocytic cells were strongly and diffusely positive for cytokeratin AE1/AE3, p63 and p40, and uniformly negative for androgen receptor, GATA3, S100, SOX10 and Her-2. A *MAML2* rearrangement was identified by FISH, thus confirming the diagnosis of oncocytic variant of mucoepidermoid carcinoma.

**Conclusion:**

In this illustrative example, we present the clinicoradiologic, cytologic, histopathologic, and immunophenotypic characteristics of this rare variant of mucoepidermoid carcinoma, together with molecular documentation.

Mucoepidermoid carcinoma (MEC) represents the most common salivary gland (SG) malignancy accounting for 2.8–16% of all SG neoplasms and 12–30% of SG carcinomas, respectively. Historically, MEC is defined as a “triphasic” tumor comprising mucous, epidermoid (squamoid) and intermediate cells [1]. However, the advent of molecular testing leading to the discovery of *MAML2::CRTC1/3* fusions in most SG MECs (40–90%) has markedly expanded the histomorphologic repertoire of MEC highlighting variants that clearly defy the existing dogma. Newly-recognized MEC variants include oncocytic (OMEC), Warthin-like, sclerosing, spindle cell and mucoacinar subtypes [1, 2]. Furthermore, adding to the level of diagnostic complexity, a fraction of MECs may be lacking any evidence of squamoid differentiation by means of light microscopy and ancillary staining [3, 4].

A 30-year-old man with unremarkable medical history presented clinically with asymptomatic, unilateral swelling of the left parotid gland. CT imaging with contrast revealed a multilobulated left parotid mass measuring 5.2 × 4.1 × 2.4 cm causing thinning of the overlying skin (Fig. 1A and B). Additionally, numerous enlarged left level 2B, 3 and 4 lymph nodes were noted, suspicious for metastasis. FNA cytopathology of the mass showed loose clusters of large cells displaying increased nuclear-to-cytoplasmic ratios and ample granular oncocytic cytoplasm (Fig. 1C), and was interpreted as an oncocytic neoplasm. Superficial left parotidectomy with radical resection of the cheek and cervical lymphadenectomy was performed.

**Fig. 1:**
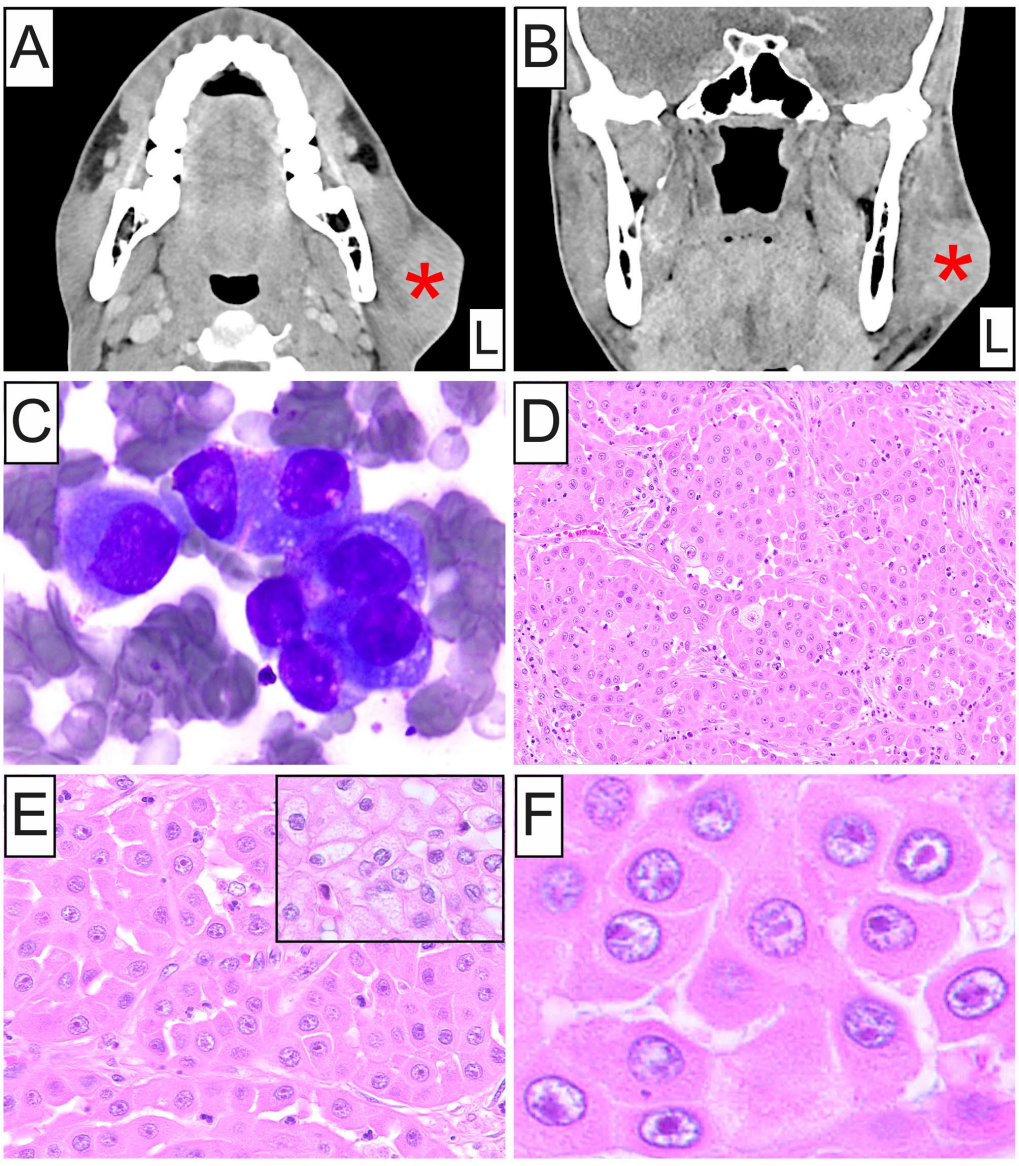
Clinical, cytopathologic and histopathologic characteristics of oncocytic mucoepidermoid carcinoma. (**A**) Axial and (**B**) coronal views of CT imaging with contrast depicting a multilobulated left parotid mass (**red asterisk**) measuring 5.2 × 4.1 × 2.4 cm causing thinning of the overlying skin. (**C**) Diff-Quik stained FNA smears of the parotid lesion showing loose clusters of large cells with increased N/C ratios and ample granular oncocytic cytoplasm. (**D**, **E**) Medium-power photomicrographs depicting infiltrative islands and nests of cohesive, polygonal, oncocytoid cells in a densely fibrous stroma. Focal oncocytic cells with cytoplasmic clearing are noted (**inset**). (**F**) High-power photomicrograph displaying lesional cells featuring enlarged, oval, open-face nuclei with coarse chromatin and a single acidophilic macronucleolus, voluminous eosinophilic granular cytoplasm and distinct cell membrane borders (**H**&**E** stain; original magnification **D**: 20x, **E**: 40x, **F**: 80x)

Histopathology disclosed a circumscribed, unencapsulated neoplasm exhibiting a solid growth pattern composed of infiltrative islands and nests of cohesive, polygonal, oncocytoid cells immersed in a densely fibrous stroma with scattered mixed inflammatory cells (Fig. 1D and **E**). Lesional cells exhibited enlarged, oval, open-face nuclei with coarse chromatin and a single acidophilic macronucleolus, voluminous eosinophilic granular cytoplasm and distinct cell membrane borders (Fig. 1E and **F**). Focal cytoplasmic clearing was also observed (Fig. 1E **inset**). Increased mitotic activity and necrosis were absent. Microcystic architecture was noted solely in a single tumor nest at the periphery (Fig. 2A). Intraluminally, cystic spaces contained eosinophilic or amphophilic mucinous secretions and were lined by cuboidal oncocytic and intermediate cells with interspersed mucin-producing cells (Fig. 2B and C), highlighted by mucicarmine stain (Fig. 2D). Immunohistochemically, oncocytic cells showed strong, diffuse positivity for cytokeratin AE1/AE3 (Fig. 3A), p63 and p40 (Fig. 3B and C), and were uniformly negative for androgen receptor (AR; Fig. 3D), GATA3, S100, SOX10 and Her-2. An underlying *MAML2* rearrangement was identified by FISH, thus confirming the diagnosis of OMEC, intermediate-grade. No evidence of metastasis was identified in any of the examined lymph nodes.

**Fig. 2:**
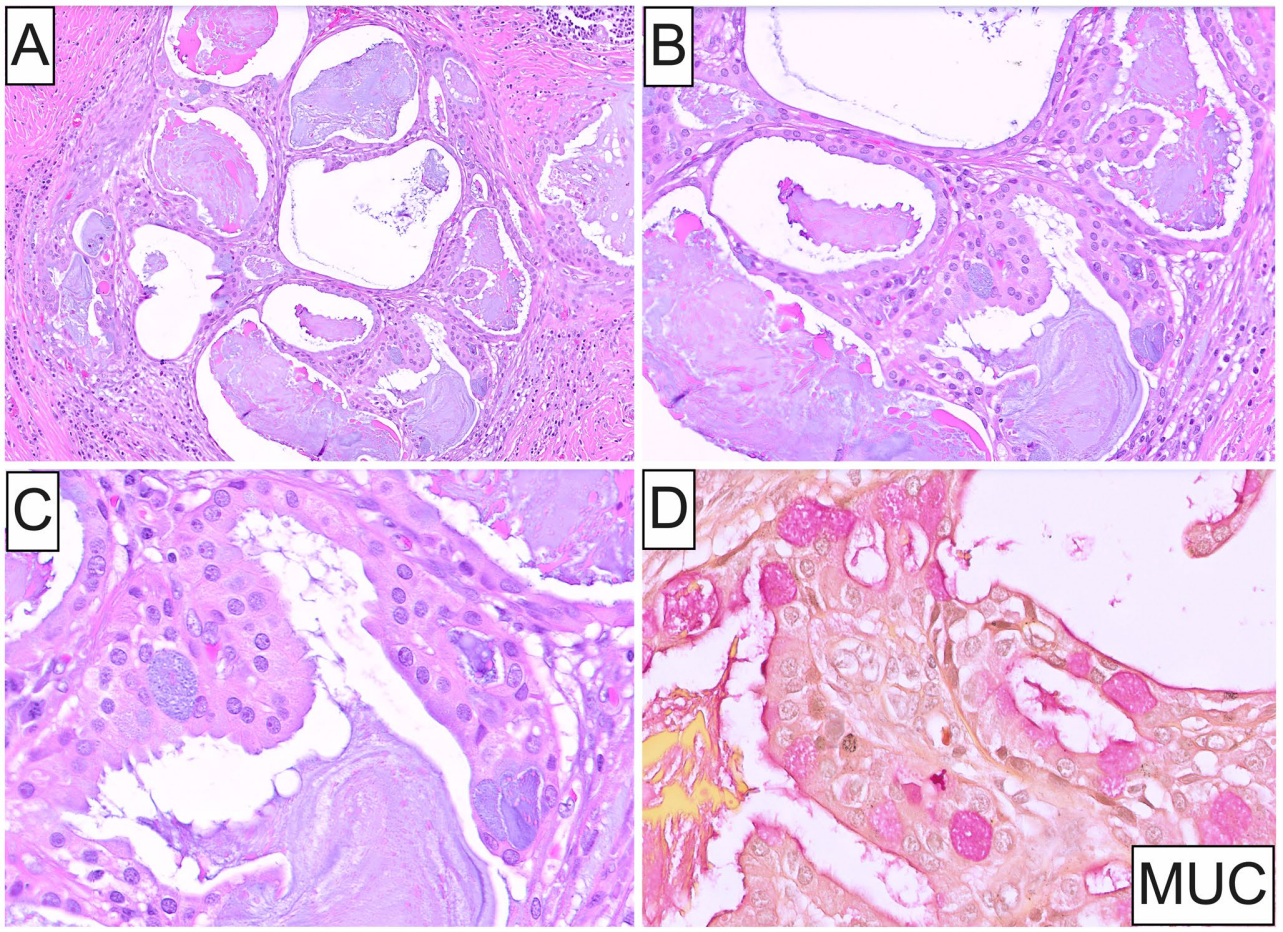
(**A**, **B**) Low- and medium-power photomicrographs depicting a single focus of classic mucoepidermoid carcinoma identified at the periphery of the tumor comprising microcystic spaces with intraluminal, eosinophilic or amphophilic, mucinous secretions. (**C**) High-power photomicrograph of the cystic spaces lined by cuboidal oncocytic and intermediate cells with interspersed mucocytes (H&E stain; original magnification **A**: 10x, **B**: 20x, **C**: 40x). (**D**) Mucicarmine stain highlighting both cytoplasmic and extracellular mucin (original magnification 40x)

**Fig. 3:**
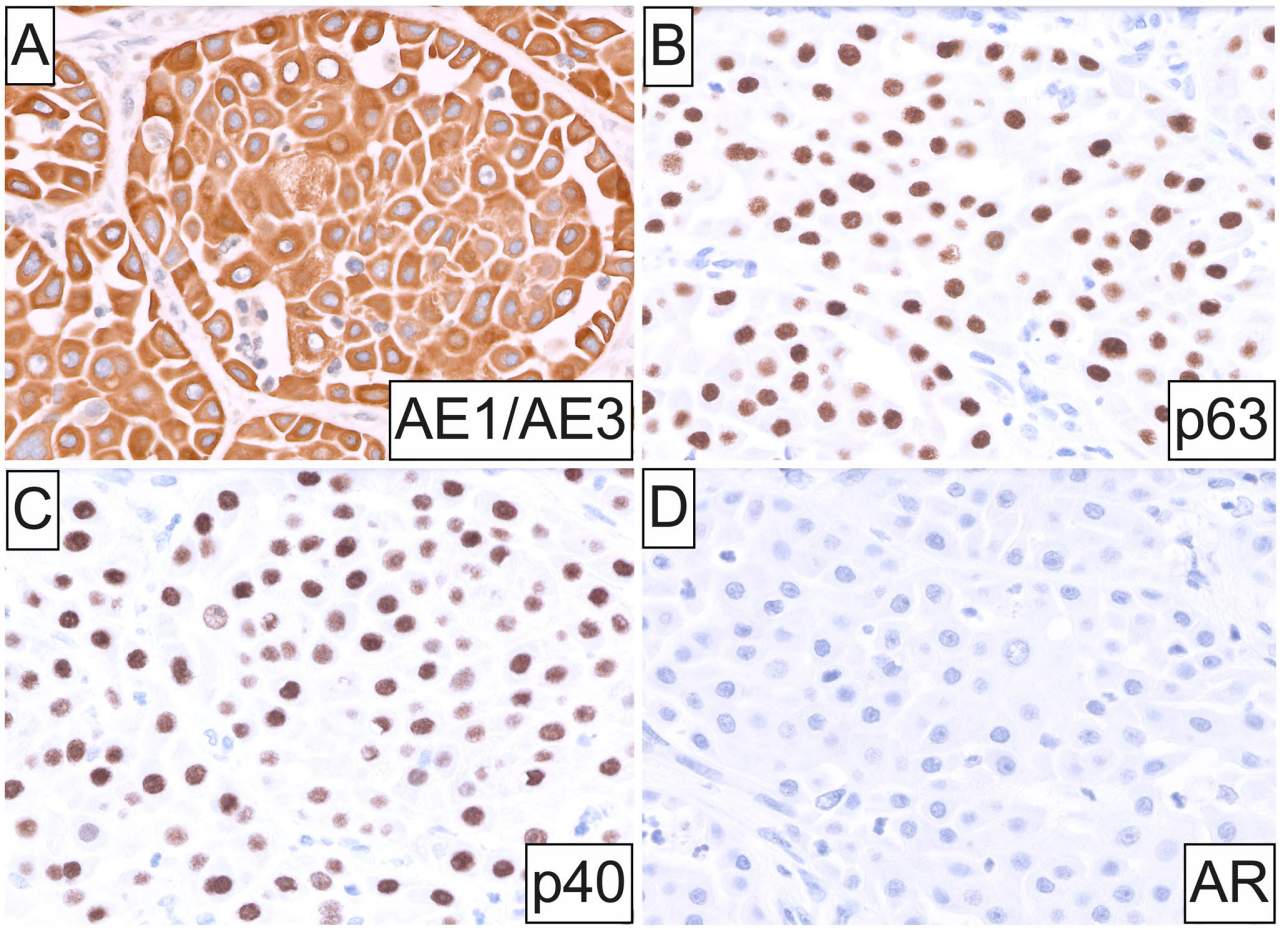
Immunophenotypic characteristics of oncocytic mucoepidermoid carcinoma. (**A**) Lesional cells showed strong, diffuse positivity for cytokeratin AE1/AE3, (**B**) p63 and (**C**) p40, and uniform negativity for (**D**) androgen receptor (original magnification **A-D**: 40x)

*Bona fide* OMEC is rare consisting almost exclusively of oncocytes, as occurred in the present case. Foci of classic MEC or mucocytes, when present, are typically sparse and not readily identifiable, especially in incisional biopsy specimens. Diagnosis of OMEC may be challenging due to significant histopathologic mimicry of other benign and malignant SG neoplasms defined by, or occasionally demonstrating, oncocytoid cytomorphology. Such lesions chiefly comprise oncocytoma and Warthin tumor, salivary duct carcinoma, secretory carcinoma, acinic cell carcinoma, as well as intraductal and so-called “oncocytic carcinoma” [2, 5]. Strong, uniform p63/p40 immunostaining in conjunction with lack of reactivity for S100/SOX10, AR and DOG1 or NOR1 may aid in discerning OMEC from its histologic mimics [1], prior to molecular confirmation. Notably, the solid growth and oncocytic phenotype observed in OMEC do not appear to impact tumor biologic behavior in most cases.

## Data Availability

No datasets were generated or analysed during the current study.
